# Ag-Doped MoSe_2_/ZnO Heterojunctions: A Highly Responsive Gas-Sensitive Material for Selective Detection of NO Based on DFT Study

**DOI:** 10.3390/nano13182510

**Published:** 2023-09-07

**Authors:** Tao He, Hongcheng Liu, Jing Zhang, Yuepeng Yang, Yuxiao Jiang, Ying Zhang, Jiaqi Feng, Kelin Hu

**Affiliations:** 1 College of Electrical Engineering, Guizhou University, Guiyang 550025, China; gs.the21@gzu.edu.cn (T.H.); zhangjing@gzu.edu.cn (J.Z.); gs.yuepengyang21@gzu.edu.cn (Y.Y.); gs.yxjiang22@gzu.edu.cn (Y.J.); zhangyingmails@126.com (Y.Z.); mm.jqfeng19@gzu.edu.cn (J.F.); 2 School of Mechanical and Electrical Engineering, University of Electronic Science and Technology of China, Chengdu 611731, China; 3 Electric Power Research Institute of China Southern Power Grid Guizhou Co., Ltd., Guiyang 550002, China

**Keywords:** density functional theory, Ag-doped MoSe_2_/ZnO heterojunctions, NO detection

## Abstract

In this work, the adsorption and sensing behavior of Ag-doped MoSe_2_/ZnO heterojunctions for H_2_, CH_4_, CO_2_, NO, CO, and C_2_H_4_ have been studied based on density functional theory (DFT). In gas adsorption analysis, the adsorption energy, adsorption distance, transfer charge, total electron density, density of states (DOS), energy band structure, frontier molecular orbital, and work function (WF) of each gas has been calculated. Furthermore, the reusability and stability of the Ag-doped MoSe_2_/ZnO heterojunctions have also been studied. The results showed that Ag-doped MoSe_2_/ZnO heterojunctions have great potential to be a candidate of highly selective and responsive gas sensors for NO detection with excellent reusability and stability.

## 1. Introduction

NO is a common atmospheric pollutant from incomplete fossil fuel combustion and vehicle exhaust emissions. It is easily ignited when in contact with flammable materials and organic matter. The brain can be harmed and paralyzed when the human body inhales a certain amount of NO [1,2]. Therefore, it is essential to carry out effective and selective NO detection to protect both the environment and human health [3,4]. A metal oxide semiconductor gas sensor has the advantages of simple operation, small size, good performance, and low cost, and is commonly used in NO gas detection [5,6]. As a typical N-type semiconductor metal material, the ZnO-based gas sensor has the characteristics of high sensitivity, simple preparation, and low synthesis cost. ZnO monolayer is a graphene-like material, which has been predicted to have more interesting electronic and magnetic properties than bulk structures [7]. In addition, previous studies have shown that ZnO monolayers have been successfully prepared [8,9]. But there are also problems of high working temperatures and poor selectivity [10,11]. The composite method of nanomaterials provides an effective way to solve the above issues [12,13].

Common methods to improve gas sensing properties of ZnO include heterojunctions construction and precious metal doping [14]. Due to its two-dimensional structure and excellent electronic transport properties, MoSe_2_ has been widely researched in the literature. MoSe_2_ has been applied in many fields such as energy storage, optoelectronics, sensors, catalysis, etc. [15,16,17]. It has been proved that the gas sensing properties can be effectively enhanced when the heterojunctions of MoSe_2_/ZnO were constructed. For example, Ravindra Kumar Jha [18] reported that 2D-MoSe_2_/0D-ZnO nanocomposites have a lower detection limit and good signal-to-noise ratio for hydrogen sulfide detection in dry air environments compared to MoSe_2_ or ZnO alone. Using the hydrothermal method, Nikita Jain [19] synthesized a ZnO-doped MoSe_2_ nanosheet ethanol gas sensor at room temperature. The synergistic effect of MoSe_2_ nanosheet and ZnO nanorods enhanced the response to ethanol gas. In addition, it has been reported that doped metal elements in ZnO can change the electronic behavior of ZnO and effectively enhance the NO gas sensitivity of ZnO-based sensors [20,21]. Bin Qiao et al. studied the adsorption of NO molecules on an Al-doped ZnO monolayer based on first principles [22]. They found that the adsorption of NO molecules on the ZnO monolayer changed from weak physical adsorption to chemisorption, significantly improving the gas sensitivity of the ZnO monolayer to NO molecules. Yongfeng Qu et al. studied the adsorption properties of CO, NO, and NH_3_ on the ZnO monolayer modified by precious metals (Ag and Au) atoms [23]. The results showed that doping Ag and Au atoms can significantly improve the adsorption capacity of ZnO monolayer for CO and NO molecules. In conclusion, the construction of MoSe_2_/ZnO heterojunctions and ZnO-doped precious metals can improve the gas sensitivity of ZnO-based sensors. However, there is currently NO relevant report on Ag-doped MoSe_2_/ZnO heterojunctions used for detecting NO gas, so studying this structure is of innovative significance.

In this work, the sensing mechanism and adsorption characteristics of H_2_, CH_4_, CO_2_, NO, CO, and C_2_H_4_ on Ag-doped MoSe_2_/ZnO heterojunctions have been investigated based on DFT calculations. By calculating and analyzing the DOS, energy band structure, and WF of the adsorption system, it is found that Ag-doped MoSe_2_/ZnO heterojunctions have great selectivity and responsiveness to NO gas. Finally, molecular dynamics was used to simulate the stability of Ag-doped MoSe_2_/ZnO heterojunctions at 400 K. These results broaden the use of MoSe_2_/ZnO heterojunctions in the gas sensing sector and offer theoretical guidance for the fabrication of gas sensors using Ag-doped MoSe_2_/ZnO heterojunctions.

## 2. Computation Details

In this paper, spin-polarized calculations were used in the DMol^3^ module, which is based on the DFT method. The GGA-PBE functional approach was used to compute the optimization, energy, and associated features of the gas–solid interface architecture [24,25]. Double numerical polarization (DNP) is chosen as the basis group function of a linear combination of atomic orbitals [26]. In DFT-D dispersion correction, the impact of the van der Waals force is adjusted using the Grimme approach [15,27]. The electron in the core was dealt with using the DFT semi-core pseudopotential (DSPP) [28]. For geometric optimization and electronic structure calculations, the K-point sample of the Monkhorst–Pack grid was sampled to 4 × 4 × 1 in the Brillouin region. The energy tolerance accuracy, maximum force, and displacement were selected as 2 × 10^−5^ Ha, 4 × 10^−3^ Ha/Å and 5 × 10^−3^ Å, respectively [29]. A more accurate 10^−6^ Ha self-consistent loop energy, a global orbital cut-off radius of 5.0 Å, and smearing of 0.005 Ha were used to produce accurate total energy values for simulations of static electronic structures [30,31].

In this paper, ZnO (001) surface and MoSe_2_ monolayer are selected to construct the heterojunctions via stacking. In this case, the MoSe_2_ monolayer is in the lower layer of the heterojunctions, and the ZnO (001) surface is in the upper layer of the heterojunctions. The ZnO (001) and MoSe_2_ monolayers were treated with 3 × 3 × 1 supercells to ensure high computational efficiency and a low lattice mismatch rate. To prevent interactions between the periodic boundary and surrounding layers, a 25 Å vacuum layer is placed in the z-direction [32].

Calculating the binding energy of Ag-doped MoSe_2_/ZnO heterojunctions is required to screen the most stable structure. The higher the binding energy, the more stable the system. Equation (1) calculates the binding energy (*E_b_*) as follows [33]:

(1) $$E_{b}{=E}_{{A\mathrm{g-}MoSe}_{2}/ZnO\;}-{\;E}_{{\mathrm{MoSe}}_{2}/\mathrm{ZnO}}-{\;E}_{\mathrm{Ag}}$$

where $E_{{\mathrm{Ag-}\mathrm{MoSe}}_{2}/\mathrm{ZnO}}$ is the doping system’s overall energy, and $E_{{\mathrm{MoSe}}_{2}/\mathrm{ZnO}}$ and $E_{\mathrm{Ag}}$ are, respectively, the energy of MoSe_2_/ZnO heterojunctions and single Ag atom.

The adsorption energy (*E_ads_*) of gases on Ag-doped MoSe_2_/ZnO heterojunctions are calculated as shown in Equation (2) [34]:

(2) $$E_{\mathrm{ads}}{=E}_{{\mathrm{Ag-}\mathrm{MoSe}}_{2}/\mathrm{ZnO}/\mathrm{gas}}\;{-\;E}_{{\mathrm{Ag-}\mathrm{MoSe}}_{2}/\mathrm{ZnO}}\;{-\;E}_{\mathrm{Gas}}$$

$E_{{\mathrm{Ag-}\mathrm{MoSe}}_{2}/\mathrm{ZnO}/\mathrm{gas}}$ represents the total energy of the adsorption system, while $E_{{\mathrm{Ag-}\mathrm{MoSe}}_{2}/\mathrm{ZnO}}$ and $E_{\mathrm{Gas}}$ represent the energy of the adsorbed substrate and individual gas molecules, respectively.

The Hirshfeld population analysis method was employed to investigate the charge transfer between gas molecules and Ag-doped MoSe_2_/ZnO heterojunctions. The transfer of charge (Q_T_) refers to the variation in the charge by the gas molecules before and after their adsorption on Ag-doped MoSe_2_/ZnO heterojunctions [35].

## 3. Results and Discussion

### 3.1. Establishment of MoSe_2_/ZnO Heterojunctions

**Figure 1:**
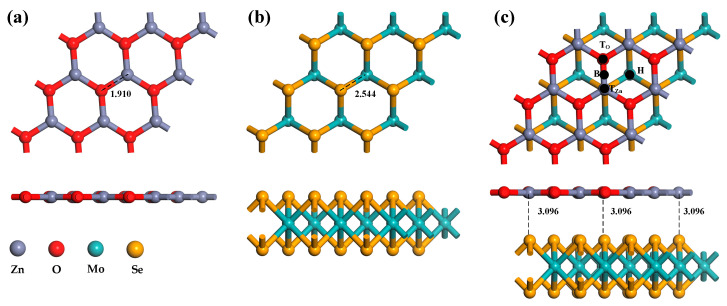
Optimized configurations of (**a**) ZnO monolayer, (**b**) MoSe_2_ monolayer, (**c**) MoSe_2_/ZnO heterojunctions.

Firstly, the super-cellular structures of ZnO and MoSe_2_ were optimized, and the optimized systems are shown in Figure 1a,b. ZnO has a graphene-like single-layer planar structure with a Zn-O bond length of 1.910 Å and a lattice length of 9.7478 Å. The Se-Mo bond length of MoSe_2_ is 2.544 Å, and the lattice length is 9.9668 Å. These parameters are consistent with the results of previous researchers, indicating that the structural optimization is successful [36,37]. Before constructing heterojunctions, the problem of lattice mismatches of less than 5% should be considered. The matching degree of lattice parameters is shown in Table 1. It can be seen from the table that the mismatch rate of lattice parameters is all less than 5%. In addition, the binding energy between MoSe_2_ and ZnO reaches −1.95 eV, which belongs to the exothermic reaction, indicating that MoSe_2_/ZnO heterojunctions have been successfully constructed. It can be seen from Figure 1c that the structural morphology of the ZnO monolayer and the MoSe_2_ monolayer in heterojunction has almost no change, and the distance between them is 3.096 Å.

**Figure 2:**
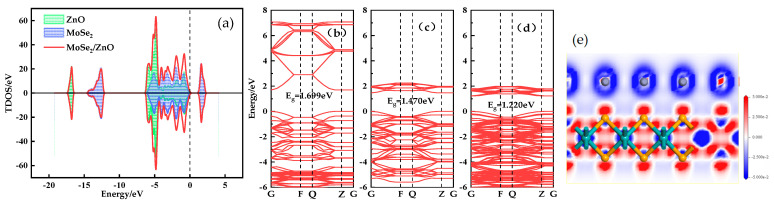
(**a**) TDOS of MoSe_2_/ZnO heterojunctions, (**b**–**d**) band structures of ZnO, MoSe_2_ and MoSe_2_/ZnO heterojunctions, and (**e**) deformation charge density maps of MoSe_2_/ZnO heterojunctions.

The TDOS of the MoSe_2_/ZnO heterojunctions is shown in Figure 2a, where there are resonance peaks and a large overlap between the DOS of ZnO and MoSe_2_ from −6.4 eV to 0 eV, which once again proves that the MoSe_2_/ZnO heterojunctions were successfully constructed. Moreover, near the Fermi energy level, the DOS peaks of MoSe_2_ are larger than those of ZnO, especially at 1.6 eV, which indicates that the electronic nature of the heterojunction is dominated by MoSe_2_. Figure 2b–d represents the energy band structure diagrams of ZnO, MoSe_2_, and MoSe_2_/ZnO heterojunctions, respectively. From the figure, it can be seen that the electron energy of ZnO in the conduction band is significantly higher than that of MoSe_2_. Therefore, when constructing the MoSe_2_/ZnO heterojunctions, the electrons at the higher energy levels in ZnO will move to the lower energy levels of MoSe_2_. As shown in Figure 2d, the movement of electrons in the ZnO monolayer to the lower energy level leads to a denser number of conduction bands in the heterojunction between 1 eV and 2 eV, and the superposition of the ZnO and MoSe_2_ valence bands leads to a denser valence band in the heterojunction. Figure 2e shows the differential charge density plot of MoSe_2_/ZnO heterojunctions, with the blue color representing the electron depletion region and the red color representing the electron concentration region. ZnO is located in the blue region, and MoSe_2_ is located in the red region, which proves the movement of electrons from ZnO to MoSe_2_. The movement of electrons leads to the formation of a built-in electric field in the MoSe_2_/ZnO heterojunctions.

### 3.2. Structural Optimization of Ag-MoSe_2_/ZnO Heterojunctions and Six Gas Molecules

**Figure 3:**
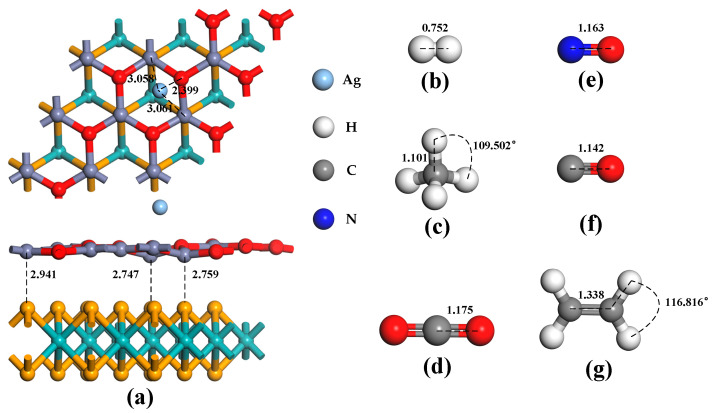
Optimized configurations of (**a**) Ag-MoSe_2_/ZnO heterojunctions, (**b**) H_2_, (**c**) CH_4_, (**d**) CO_2_, (**e**) NO, (**f**) CO, and (**g**) C_2_H_4_.

The Ag atom has four doping sites on the ZnO monolayer in the heterojunctions, as illustrated in Figure 1c: T_Zn_, T_O_, B, and H. When the binding energies of these four doping methods are compared, the absolute binding energy at the H site is found to be the highest, at −0.94 eV. As a result, the structure of the doping mode is chosen for the subsequent computation of gas molecule adsorption. As shown in Figure 3a, the ZnO monolayer in the heterojunctions is distorted to a certain extent after Ag atom doping. In addition, the distance between the two monolayer structures of the MoSe_2_/ZnO heterojunctions is shortened. These indicate that Ag atoms interact with heterojunctions. Figure 3b–g show the optimized bond lengths and bond angles of H_2_, CH_4_, CO_2_, NO, CO, and C_2_H_4_, respectively, consistent with the optimization results of these gas molecules by previous researchers [22,36,38,39].

**Figure 4:**
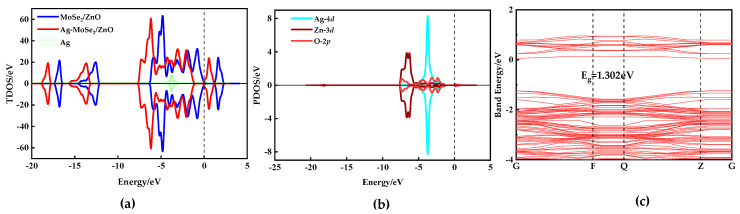
(**a**) TDOS of MoSe_2_/ZnO heterojunctions pre- and post-Ag doping, (**b**) PDOS of the Ag-MoSe_2_/ZnO heterojunctions, and (**c**) band structures of Ag-MoSe_2_/ZnO heterojunctions.

In the total density of states (TDOS) diagram shown in Figure 4a, the spin-up and spin-down TDOS curves are entirely symmetric, indicating that both MoSe_2_/ZnO heterojunctions and Ag-MoSe_2_/ZnO heterojunctions are non-magnetic. The black dashed line at zero represents the Fermi level. Compared with the MoSe_2_/ZnO heterojunctions, the TDOS curves of the Ag-MoSe_2_/ZnO heterojunctions are significantly shifted to the left toward lower energy. Meanwhile, the Fermi energy level is closer to the conduction band. As shown in the partial density of states (PDOS) curves in Figure 4b, the 4*d* orbitals of Ag overlap with the 3*d* orbitals of Zn and the 2*p* orbitals of O near −7 eV and between −5 eV and −1 eV, which suggests that the Ag atoms interact violently with the monolayer of ZnO in the heterojunctions. Figure 4c shows the energy band structure of the Ag-MoSe_2_/ZnO heterojunctions. The gap between the valence band and the conduction band is 1.302 eV, and the conduction band is closer to the Fermi energy level at zero, which is consistent with the analysis of the previous TDOS diagram.

### 3.3. Adsorption Properties of Six Gas Molecules on Ag-MoSe_2_/ZnO Heterojunctions

**Figure 5:**
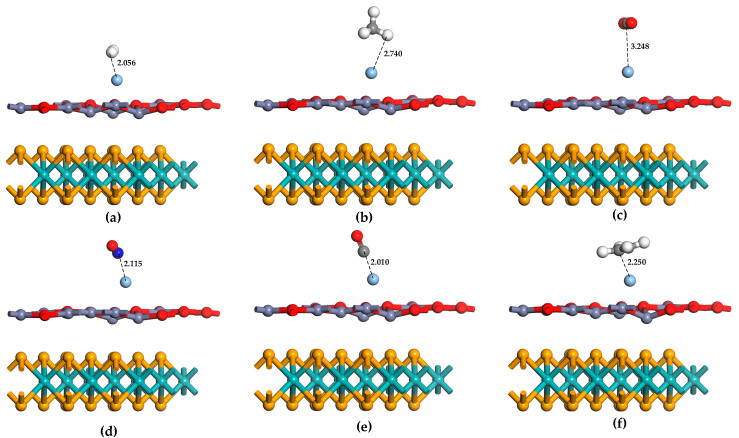
The most stable structure of six gas molecules adsorbed on Ag-MoSe_2_/ZnO heterojunctions: (**a**) H_2_, (**b**) CH_4_, (**c**) CO_2_, (**d**) NO, (**e**) CO, (**f**) C_2_H_4_.

In this work, these six gas molecules are considered to approach the Ag-MoSe_2_/ZnO heterojunctions with different orientations and angles, and the structure of each adsorption configuration is optimized. The optimization results were then analyzed, and the adsorption system configurations of the six gases with the largest absolute values of adsorption energies were selected, as shown in Figure 5. The characteristic and geometric parameters of the six gas adsorption systems are shown in Table 2. From these parameters, it can be seen that the adsorption performance of NO, CO, and C_2_H_4_ by Ag-MoSe_2_/ZnO heterojunctions is relatively ideal, and the adsorption energy is −1.06 eV, −1.01 eV, and −1.07 eV, respectively. This is chemisorption. The bond length and bond angle of NO, CO, and C_2_H_4_ changed significantly before and after adsorption, indicating that these gas molecules were activated after capture. Ag-MoSe_2_/ZnO heterojunctions have a poor adsorption effect on H_2_, CH_4_, and CO_2_, with adsorption energies of −0.05 eV, −0.16 eV, and −0.09 eV. Furthermore, the bond lengths and bond angles of the gas molecules change less before and after the adsorption of CH_4_ and CO_2_, and the adsorption distances are larger. The low probability of CH_4_ and CO_2_ adsorption on the Ag-MoSe_2_/ZnO heterojunctions was demonstrated again. Interestingly, the adsorption energy of H_2_ is low, but the adsorption distance is short. Physical adsorption dominated by van der Waals forces is considered to be the primary interaction between H_2_ and Ag-MoSe_2_/ZnO heterojunctions. The Q in Table 2 shows that H_2_, CH_4_, CO, and C_2_H_4_ transfer electrons to the Ag-MoSe_2_/ZnO heterojunctions, and NO and CO_2_ gain electrons from the Ag-MoSe_2_/ZnO heterojunctions. Among them, NO has the largest Q. In contrast, CH_4_ and CO_2_ have negligible Q.

**Figure 6:**
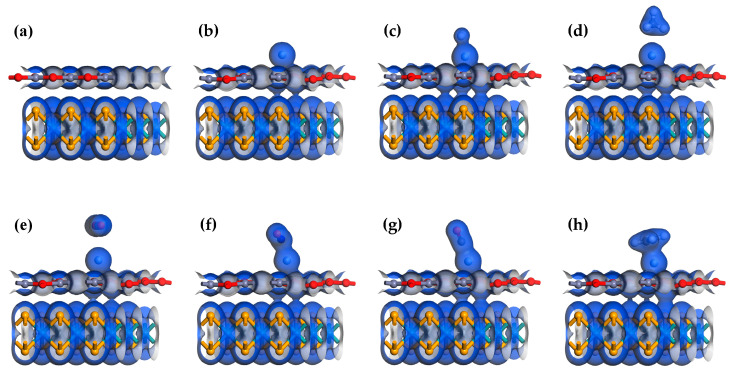
Total electron density of (**a**) MoSe_2_/ZnO heterojunctions, (**b**) Ag-MoSe_2_/ZnO heterojunctions, (**c**) Ag-MoSe_2_/ZnO/H_2_ system, (**d**) Ag-MoSe_2_/ZnO/CH_4_ system, (**e**) Ag-MoSe_2_/ZnO/CO_2_ system, (**f**) Ag-MoSe_2_/ZnO/NO system, (**g**) Ag-MoSe_2_/ZnO/CO system, and (**h**) Ag-MoSe_2_/ZnO/C_2_H_4_ system. Isosurface value: 0.2 e/Å^3^.

The adsorption energy analysis of these six gases does not indicate that Ag-MoSe_2_/ZnO heterojunctions are highly selective to NO. Therefore, further analysis is needed. Figure 5 shows the total electron density maps of the MoSe_2_/ZnO heterojunctions, Ag-MoSe_2_/ZnO heterojunctions, and six gas adsorption systems. Figure 6a shows that in the heterojunction, the electron exchange between MoSe_2_ and ZnO monolayers is weak. As shown in Figure 6b, after the addition of the Ag atom dopant, the ZnO monolayer communicates electronically with the MoSe_2_ monolayer, increasing the connection between the two layers. As shown in Figure 6d–h, there is almost no electron exchange between CH_4_ and CO_2_ and the Ag-MoSe_2_/ZnO heterojunctions, which is in agreement with the analyzed results of Q. The total electron density of CH_4_ and CO_2_ was found to be very small. The strongest interaction between NO and Ag-MoSe_2_/ZnO heterojunctions can be found by analyzing Q and total electron density.

### 3.4. DOS Analysis of Six Gas Adsorption Systems

**Figure 7:**
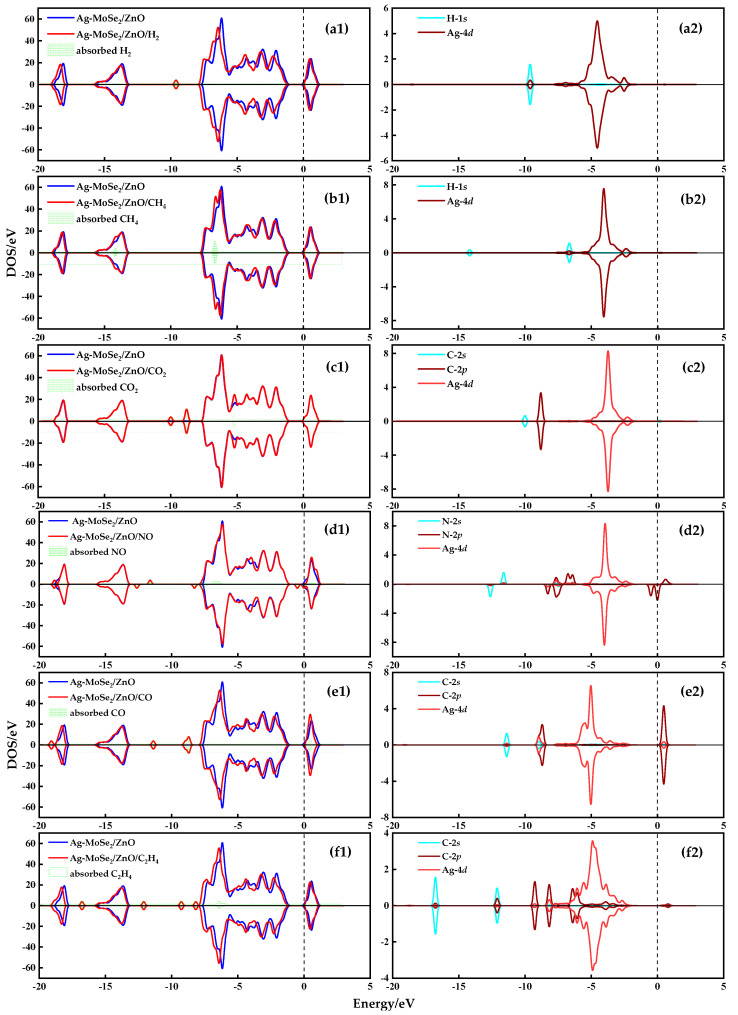
TDOS and PDOS of different gas systems (**a**) Ag-MoSe_2_/ZnO/H_2_ system, (**b**) Ag-MoSe_2_/ZnO/CH_4_ system, (**c**) Ag-MoSe_2_/ZnO/CO_2_ system, (**d**) Ag-MoSe_2_/ZnO/NO system, (**e**) Ag-MoSe_2_/ZnO/CO system, and (**f**) Ag-MoSe_2_/ZnO/C_2_H_4_ system.

To further investigate the high selectivity of Ag-MoSe_2_/ZnO heterojunctions for NO, the DOS plots of the most stable adsorption configurations of these six gases on Ag-MoSe_2_/ZnO heterojunctions were analyzed. In Figure 7, the left is the TDOS diagram, and the right is the PDOS diagram. As shown in Figure 7(a1–f1), the TDOS curves of the adsorbed systems of H_2_, CH_4_, NO, CO, and C_2_H_4_ are significantly different before and after adsorption, and all of them are shifted to the low-energy direction. This indicates that the Ag-MoSe_2_/ZnO heterojunctions are more stable after adsorption by H_2_, CH_4_, NO, CO, and C_2_H_4_. However, the TDOS curve of the CO_2_ adsorption system has little change before and after adsorption, proving that the interaction between Ag-MoSe_2_/ZnO heterojunctions and CO_2_ is very weak. By comparing the TDOS plots of these gas molecules, it can be seen that only the NO adsorption system has new electronic states at the Fermi energy level. These new electronic states lead to a narrowing of the intercept of the curve near the Fermi energy level, which implies that the adsorption of NO gas reduces the band gap of the system. Because there is an inverse relationship between conductivity and bandgap, changes in bandgap provide the theoretical basis for generating electrical signals [40]. In addition, the TDOS diagram of the NO adsorption system is not symmetric, indicating that the adsorption system is magnetic with a magnetic moment of 1.48 μ_B_. The asymmetry of the TDOS diagram near the Fermi level can be explained by the 2*p* orbital of N in the PDOS curve in Figure 7(d2).

### 3.5. Analysis of Energy Band Structure of Six Gas Adsorption Systems

**Figure 8:**
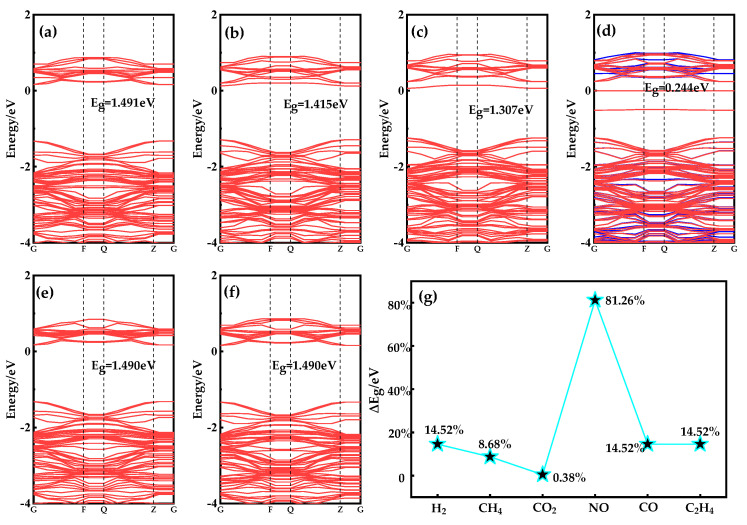
Band structures of (**a**) H_2_ system, (**b**) CH_4_ system, (**c**) CO_2_ system, (**d**) NO system, (**e**) CO system, (**f**) C_2_H_4_ system, and (**g**) ΔE_g_ of Ag-MoSe_2_/ZnO heterojunctions after gas adsorption.

To further verify that only NO among the six gases can significantly reduce the band gap of the adsorption system, the energy band structures of the adsorption systems of these six gases are analyzed in Figure 8. The change in *E_g_* is useful in determining whether the conductivity of an adsorption system has increased or decreased. To calculate the detection sensitivity of Ag-doped MoSe_2_/ZnO heterojunctions for six gases (change in gas sensor resistance), Formulas (3) and (4) can be used to calculate the electrical conductivity (*σ*) and sensitivity (*S*) of an Ag-doped MoSe_2_/ZnO heterojunctions/gas adsorption system:

(3) $$\sigma \propto {\;\exp(-E}_{g}/2\mathrm{KT})$$

(4) $${S=|(\sigma }_{{\mathrm{Ag-}\mathrm{MoSe}}_{2}/\mathrm{ZnO}/\mathrm{Gas}\;}{\;-\;\sigma }_{{\mathrm{Ag-}\mathrm{MoSe}}_{2}/\mathrm{ZnO}}{)|/\sigma }_{{\mathrm{Ag-}\mathrm{MoSe}}_{2}/\mathrm{ZnO}\;}$$

where *K* is the Boltzmann constant and *T* is the working temperature, and $\sigma_{{\mathrm{Ag-}\mathrm{MoSe}}_{2}/\mathrm{ZnO}/\mathrm{Gas}\;}$ and $\sigma_{{\mathrm{Ag-}\mathrm{MoSe}}_{2}/\mathrm{ZnO}}$ are the electrical conductivity of a gas adsorption system and Ag-doped MoSe_2_/ZnO heterojunctions, respectively [41]. It can be known from Formula (3) that *σ* of the adsorption system is inversely proportional to *E_g_*. After knowing the relationship between *σ* and *E_g_* and combining it with Formula (4), the sensitivity of gas molecules adsorbed on Ag-doped MoSe_2_/ZnO heterojunctions can be calculated.

As can be seen from Figure 4c, the E_g_ of Ag-doped MoSe_2_/ZnO heterojunction is 1.302 eV. Combined with the data in Figure 8a–f, it is calculated that the ΔE_g_ after adsorption of H_2_, CH_4_, CO_2_, NO, CO, and C_2_H_4_ by Ag-doped MoSe_2_/ZnO heterojunctions is 0.189 eV, 0.113 eV, 0.005 eV, 1.058 eV, 0.188 eV, and 0.188 eV, respectively. The ΔE_g_ after NO adsorption is the largest, which is 1.058 eV. The results show that Ag-doped MoSe_2_/ZnO heterojunction conductivity changes the most after NO adsorption. In addition, only NO increases the conductivity of Ag-doped MoSe_2_/ZnO heterojunctions after adsorption, while the other five gases decrease. The vast difference in conductivity changes lays a theoretical foundation for the high selection of NO gas sensors. Figure 8g shows the sensitivity of the Ag-doped MoSe_2_/ZnO heterojunctions to these six gases, in which NO sensitivity is as high as 81.26%. The maximum sensitivity of the remaining five gases does not exceed 15%. In summary, Ag-doped MoSe_2_/ZnO heterojunctions have high selectivity and response to NO in the specific environment of these six gases.

### 3.6. Work Function Analysis of Six Gas Adsorption Systems

**Figure 9:**
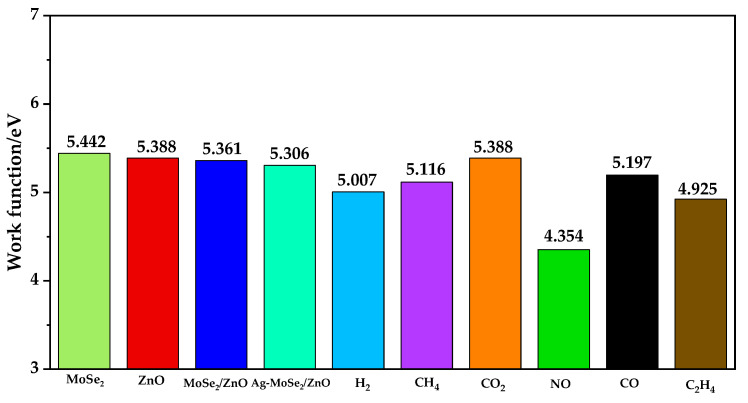
WF of ZnO, MoSe_2_, ZnO/MoSe_2_ heterojunctions, Ag-MoSe_2_/ZnO heterojunctions, and six gas adsorption systems.

The work function is the minimum energy required to transfer electrons from the surface to the vacuum layer [42]. Considering that the work function of the material surface is susceptible to the adsorption behavior of gases, Figure 9 calculates work functions for ZnO, MoSe_2_, ZnO/MoSe_2_ heterojunctions, Ag-doped MoSe_2_/ZnO heterojunctions, and six gas adsorption systems. From the figure, it can be seen that the WF of MoSe_2_ is larger than that of ZnO, which again proves that the electrons will be transferred from ZnO to MoSe_2_ in the MoSe_2_/ZnO heterojunction. The WF on the Ag-doped MoSe_2_/ZnO heterojunctions surface increases only after the adsorption of CO_2_ gas molecules, while the other five kinds of WF decrease. It indicates that CO_2_ gas molecules hinder the electron migration on the surface of Ag-doped MoSe_2_/ZnO heterojunctions, and the other five gases all promote the electron jump on the surface of Ag-doped MoSe_2_/ZnO heterojunctions to different degrees. The ΔWF induced by the adsorption of H_2_, CH_4_, CO_2_, NO, CO, and C_2_H_4_ were calculated to be 0.299 eV, 0.190 eV, 0.082 eV, 0.952 eV, 0.109 eV, and 0.381 eV, respectively. It can be seen that ΔWF is minimized after the adsorption of CO_2_, which corresponds to its poor adsorption performance in Ag-doped MoSe_2_/ZnO heterojunctions, while after the adsorption of NO, ΔWF is the largest at 0.952 eV, significantly reducing the work function on the surface of Ag-doped MoSe_2_/ZnO heterojunctions. This indicates that the Ag-doped MoSe_2_/ZnO heterojunctions are very sensitive to NO and have high selectivity.

### 3.7. Research on Energy Bands Based on the Frontline Orbit Theory

**Figure 10:**
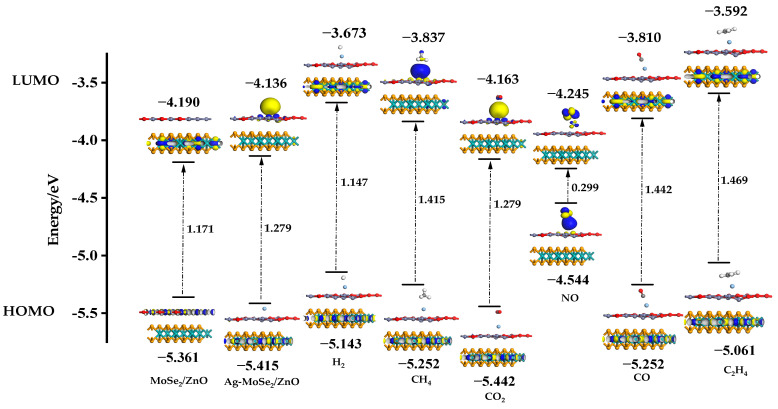
Distribution of HOMO and LUMO on ZnO/MoSe_2_, Ag-MoSe_2_/ZnO, and six gas adsorption systems.

To further analyze the interaction of Ag-doped MoSe_2_/ZnO heterojunctions with the six gases, we analyzed their HOMO (Highest Occupied Molecular Orbital) and LUMO (Lowest Unoccupied Molecular Orbital) levels. The distributions of HOMO and LUMO on ZnO/MoSe_2_, Ag-MoSe_2_/ZnO, and six gas adsorption systems are shown in Figure 10. It can be seen from the figure that the HOMO of ZnO/MoSe_2_ heterojunctions is mainly distributed on ZnO, and the LUMO is mainly distributed on MoSe_2_. After the ZnO/MoSe_2_ heterojunctions were doped with Ag atoms, the distribution of HOMO and LUMO changed significantly, with LUMO mainly distributed around Ag atoms and HOMO distributed on MoSe_2_. This may be due to the electron redistribution in the heterojunctions caused by the doping of Ag atoms. In addition, the unique LUMO distribution around Ag atoms provides active sites for the adsorption of gas molecules. Observation of the distribution of HOMO and LUMO in these six gas adsorption systems reveals that most of the HOMO and LUMO are distributed on MoSe_2_, which again demonstrates that the electronic nature of MoSe_2_/ZnO heterojunctions is mainly dominated by MoSe_2_. In addition, only NO molecules are distributed with a large amount of HOMO and LUMO, which indicates that NO molecules are strongly adsorbed with Ag-doped MoSe_2_/ZnO heterojunctions. Based on the HOMO and LUMO energies of each system, the band gaps of the different systems can be calculated. The band gap of the ZnO/MoSe_2_ heterojunctions increases slightly after doping with Ag atoms. It can be found that there is an extremely significant decrease in the band gap of the adsorption system after the adsorption of NO, whereas the band gap of the adsorption system changes less for the adsorption of the other five gases. The high selectivity of Ag-doped MoSe_2_/ZnO heterojunctions for NO was again demonstrated through the analysis of HOMO and LUMO.

### 3.8. Reusability and Stability Analysis of Ag-MoSe_2_/ZnO Heterojunctions

In the practical application of gas sensors, the reusability of the gas-sensitive material must be considered. Therefore, the calculation and analysis of the desorption time is crucial. Equation (5) is usually used to calculate the desorption time [43].

(5) $${\tau =A}^{-1}e^{\frac{-E_{a}}{k_{B}T}}$$

where *E_a_* is the chemical bond energy that must be overcome by desorption, which is assumed to be equal in value to *E_ads_*, *T* is the temperature, *k_B_* is the Boltzmann constant, and *A* is the attempt frequency (10^12^ s^−1^).

**Figure 11:**
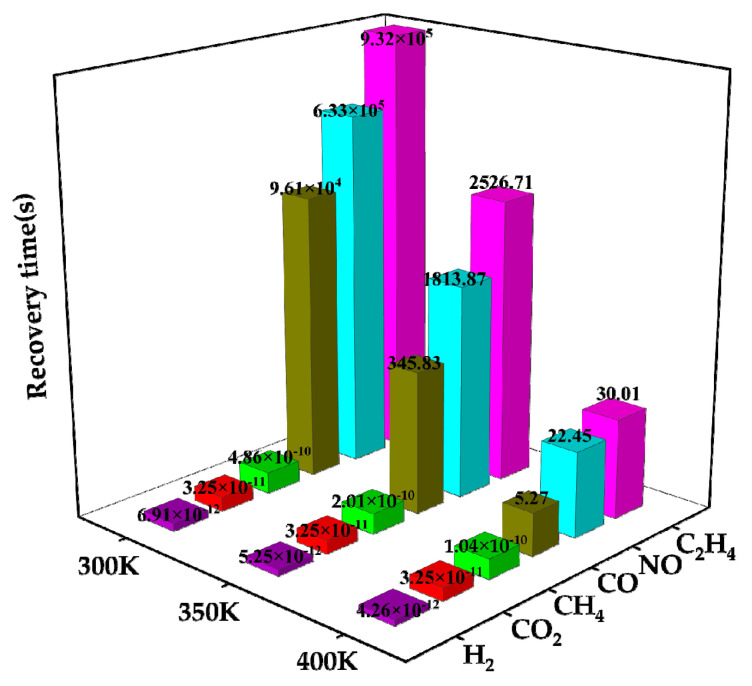
Time taken by six gases to desorb from Ag-MoSe_2_/ZnO heterojunctions at various temperatures.

The desorption times of these six gases on Ag-MoSe_2_/ZnO heterojunctions at three different temperatures are analyzed in Figure 11. The graph demonstrates that as the temperature rises, the desorption time reduces. At 400 K, the H_2_, CH_4_, and CO_2_ desorption time is particularly small, corresponding to their small adsorption energy. CO, NO, and C_2_H_4_ have excellent desorption time at 400 K. Previous studies have repeatedly demonstrated that Ag-MoSe_2_/ZnO heterojunctions pairs have high selectivity for NO, so the analysis in this part mainly focuses on NO molecules. As can be seen from Figure 11, at 400 K temperature, the NO response recovery time is 22.45 s, indicating that short desorption can be achieved at low power consumption. Therefore, Ag-MoSe_2_/ZnO heterojunctions can be used as a low-power, reusable, highly selective, and highly responsive gas-sensitive material for NO detection.

**Figure 12:**
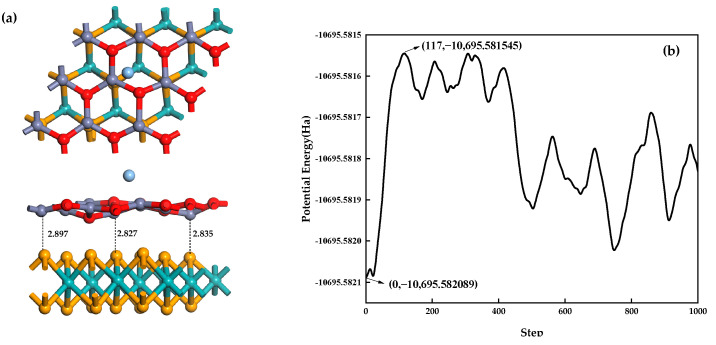
(**a**) Structure diagram of Ag-MoSe_2_/ZnO heterojunctions after first-principles molecular dynamics simulation; (**b**) potential energy fluctuation curve of Ag-MoSe_2_/ZnO heterojunctions.

Based on the first-principles molecular dynamics theory, Ag-MoSe_2_/ZnO heterojunction stability is analyzed. This paper used an NVT (dynamics at fixed volume with a thermostat to maintain a constant temperature) ensemble to simulate Ag-MoSe_2_/ZnO heterojunctions at 400 K with a duration of 1 ps, a time step of 1 fs, and a step number of 1000 [44]. Figure 12a shows the structure diagram of Ag-MoSe_2_/ZnO heterojunctions after first-principle molecular dynamics simulation. It can be seen from the graph that although Ag atoms and some oxygen atoms on the ZnO monolayer move significantly, in general, the distance between the ZnO monolayer and MoSe_2_ monolayer is reduced, and the heterojunctions are more stable. The total potential energy fluctuation curve for heterojunctions during the simulation process is depicted in Figure 12b. It can be seen from the potential energy fluctuation curve that the total potential energy of the heterojunctions fluctuates in the range of −10,695.582089~−10,695.581545 Ha during the simulation, with a range of 0.000544 Ha, which proves that the energy of the heterojunctions is relatively stable. In summary, Ag-MoSe_2_/ZnO heterojunctions can exist stably at 400 K.

## 4. Conclusions

In this work, MoSe_2_/ZnO heterojunctions have been constructed via stacking. The lattice mismatch rate and binding energy of the heterojunctions has been calculated, which proved that the structure of MoSe_2_/ZnO heterojunctions was rational. Then, the Ag-MoSe_2_/ZnO heterojunctions model was constructed, and the adsorption properties of H_2_, CH_4_, CO_2_, NO, CO, and C_2_H_4_ were calculated based on the model. The results show that Ag-MoSe_2_/ZnO heterojunctions have suitable adsorption energy for NO and a much higher Q than the other five gases. In addition, the band gap and work function of Ag-MoSe_2_/ZnO heterojunctions are very sensitive to NO, but not to H_2_, CH_4_, CO_2_, CO, and C_2_H_4_. After NO adsorption at Ag-MoSe_2_/ZnO heterojunctions, the band gap decreases from 1.302 eV to 0.244 eV, and the work function decreases from 5.306 eV to 4.354 eV. Finally, the molecular dynamics simulation results of the Ag-MoSe_2_/ZnO heterojunctions show that the heterojunctions can exist stably at 400 K. In conclusion, Ag-MoSe_2_/ZnO heterojunctions can be used as candidate materials for NO sensors with high selectivity and responsiveness.

## Figures and Tables

**Table 1:** Statistical table of lattice mismatch rates of MoSe_2_/ZnO heterojunctions.

	Model	MoSe_2_	ZnO	MoSe_2_/ZnO Heterojunctions	Lattice Mismatch Rate
Lattice Constant					
a		9.9668 Å	9.7478 Å	9.8573 Å	2%
b		9.9668 Å	9.7478 Å	9.8573 Å	2%
γ		120°	117°	118.5°	3%

**Table 2:** The characteristic parameters and geometric parameters of six kinds of gas adsorption systems.

Systems	E_ads_ (eV)	Q (e)	Adsorption Distance (Å)	Bond Length (Å)		Bond Angle (°)
Ag-MoSe_2_/ZnO/H_2_	−0.05	0.06	2.056	0.779 (H-H)		-
Ag-MoSe_2_/ZnO/CH_4_	−0.16	0.01	2.740	1.102 (C-H)		108.025 (H-C-H)
Ag-MoSe_2_/ZnO/CO_2_	−0.09	−0.02	3.248	1.175 (C-O)		-
Ag-MoSe_2_/ZnO/NO	−1.06	−0.13	2.115	1.195 (N-O)		-
Ag-MoSe_2_/ZnO/CO	−1.01	0.05	2.010	1.181 (C-O)		-
Ag-MoSe_2_/ZnO/C_2_H_4_	−1.07	0.09	2.250	1.380 (C-C)	1.095 (C-H)	121.248 (H-C-H)

## Data Availability

The data are available upon request from the corresponding author.
